# Porcine Beta-Defensin 2 Provides Protection Against Bacterial Infection by a Direct Bactericidal Activity and Alleviates Inflammation via Interference With the TLR4/NF-κB Pathway

**DOI:** 10.3389/fimmu.2019.01673

**Published:** 2019-07-18

**Authors:** Chao Huang, Xi Yang, Jing Huang, Xiao Liu, Xiaoyu Yang, Hui Jin, Qi Huang, Lu Li, Rui Zhou

**Affiliations:** ^1^State Key Laboratory of Agricultural Microbiology, College of Veterinary Medicine, Huazhong Agricultural University, Wuhan, China; ^2^Key Laboratory of Preventive Veterinary Medicine in Hubei Province, The Cooperative Innovation Center for Sustainable Pig Production, Wuhan, China; ^3^Key Laboratory of Pig Industry Sciences, Chongqing Academy of Animal Sciences, Chongqing, China; ^4^Key Laboratory of Development of Veterinary Diagnostic Products, Ministry of Agriculture and Rural Affairs of the People's Republic of China, Wuhan, China; ^5^International Research Center for Animal Disease, Ministry of Science and Technology of the People's Republic of China, Wuhan, China

**Keywords:** PBD-2, anti-infection, bactericidal activity, immune regulation, TLR4/NF-κB

## Abstract

Porcine beta-defensin 2 (PBD-2) which is a member of the family of antimicrobial peptides, is widely expressed in pig organs with a broad spectrum of bactericidal activities confirmed *in vitro*. We previously demonstrated that transgenic (TG) pigs overexpressing PBD-2 could resist the infection by the porcine pathogen *Actinobacillus pleuropneumoniae*. In this study, the roles of PBD-2 in protecting against bacterial infection were further investigated. The biochemical indexes of the blood sample, body weights, histological morphologies, and weights of the organs of TG mice expressing PBD-2 were measured. Results confirmed that these mice showed normal physiological features. An assay of *Salmonella* Typhimurium infection was conducted on wild-type (WT) and TG mice. The TG mice possessed higher survival rate, less body weight loss, and pathological changes and smaller recovery rates of bacteria after infection with *S*. Typhimurium. The *in vitro* synthetic PBD-2 and the serum and tissue homogenates from the TG mice displayed a direct bactericidal activity. Moreover, PBD-2 could inhibit the release of the proinflammatory cytokines, including IL-6, TNF-α, IL-1β, and IL-12, in the TG mice infected with *S*. Typhimurium or treated with lipopolysaccharide (LPS). The WT mice treated with PBD-2 and *S*. Typhimurium or LPS showed reduced levels of proinflammatory cytokines. The mouse macrophage cell line RAW 264.7 which expressed PBD-2 was constructed to detect the signal pathways affected by PBD-2. The suppressing effect of PBD-2 on the release of the proinflammatory cytokines was confirmed using RAW 264.7 either expressing PBD-2 or supplemented with PBD-2. The promoter activity and mRNA level of NF-κB were detected, and PBD-2 was shown to significantly inhibit the activation of the NF-κB pathway induced by LPS. The direct interaction of PBD-2 with TLR4 was revealed by isothermal titration calorimetry and far-Western blot *in vitro* and the coimmunoprecipitation of PBD-2 with TLR4 on RAW 264.7 cells. This interaction indicates one reason for the interference of NF-κB activation. Overall, this study showed that PBD-2 protected against bacterial infection through a direct bactericidal activity and alleviated inflammation by interfering with the TLR4/NF-κB pathway.

## Introduction

Bacterial drug resistance has become a global concern. Endeavors in search for antibiotic substitutes are desperately needed. Antimicrobial peptides (AMPs) are natural anti-infective agents identified in various species and implicated in innate immunity (1). These agents have been suggested to be a prominent alternative to antibiotics in fighting against infectious diseases because of their significant antibacterial activity (2). AMPs not only have a direct bactericidal activity but also regulate immune and inflammatory responses (3). As bactericides, cationic AMPs exert bactericidal activities by disrupting the permeability of the cell membrane, thereby causing bacterial content leakage (4). As immune regulatory molecules, AMPs can modulate both innate, and adaptive immunity (2). Many host peptides can serve as chemokines to recruit and activate immune cells, modulate the release of cytokines, suppress the apoptosis of neutrophils, and act as a link between innate and adaptive immune systems (5). Some human AMPs, including LL-37 (6), hBD-3 (7), and IDR-1 (8), can suppress inflammation by inhibiting specific inflammatory signaling pathways. The process of the immune regulatory and anti-inflammatory effects of AMPs is complicated and yet to be fully explored.

Defensins are cysteine-rich, amphipathic cationic small peptides that comprise a major subfamily of AMPs. They have been identified in reptiles, amphibians, birds and mammals (9). Three subclasses of defensins in primates are determined on the basis of the distribution of six-cysteine residues and disulfide bonds between cysteines: α-defensins are the most common subclass existing in neutrophils and Paneth cells of the small intestine; β-defensins mainly protect the skin and the mucous of the respiratory, genitourinary, and gastrointestinal tracts; and θ-defensins are expressed in primates other than humans (10).

To date, only β-defensins have been found in pigs. Porcine beta-defensin 2 (PBD-2) which shares a high sequence similarity with human β-defensin 1, has been shown to have a high and broad spectrum of bactericidal activity *in vitro* with a limited hemolytic activity (11). In pigs, PBD-2 is mainly expressed in tongue, liver, kidney, small intestine, and large intestine (12). PBD-2 can promote growth, alleviate diarrhea in piglets (13) and serve as a medicated feed additive for weaned piglets (14). The oral administration of PBD-2 can improve growth performance and caecum microbial flora and reduce the expression of inflammatory cytokines in pigs (15). Our previous study showed that overexpressing PBD-2 in transgenic (TG) pigs enhances the resistance to the infection of the important porcine pathogen *Actinobacillus pleuropneumoniae* (16). However, the anti-infection mechanism of PBD-2 *in vivo* and its roles in immune response remain largely unknown.

The Gram-negative bacterium *Salmonella enterica* serovar Typhimurium is an important foodborne pathogen that causes diseases in various animals and humans (17). After oral ingestion, the typical symptoms caused by *S*. Typhimurium include bacteraemia, fever, abdominal pain, vomiting, diarrhea, and body weight loss which results from gastroenteritis (18–20). Intestinal inflammation produced by *S*. Typhimurium is mainly caused by the persistence of the pathogen in the caecum (21). *S*. Typhimurium can also cross the intestinal epithelial cell layer which establishes systemic infections (17). *S*. Typhimurium can replicate in host macrophages, thereby stimulating the activity of NF-κB and the secretion of proinflammatory cytokines (22, 23). In this study, TG mice expressing PBD-2 were used as *in vivo* PBD-2 expression models to assess the effect of PBD-2 in protecting the animals from *S*. Typhimurium infection. The results presented that PBD-2 TG mice showed an increased resistance to bacterial infection compared with wild-type (WT) mice. PBD-2 not only exhibited a direct bactericidal activity but also alleviated inflammation caused by *S*. Typhimurium infection or LPS stimulation in mice. Toll-like receptors and signaling pathways regulated by PBD-2 were determined by using PBD-2-expressing macrophages and macrophages supplied with synthetic PBD-2. The TLR4/NF-κB signaling pathway was identified to be suppressed by PBD-2.

## Materials and Methods

### Peptides, Cell Line, Bacterial Strains, and Culture Methods

The mature peptide of PBD-2 was synthesized by Neweast Biosciences (# 60001, Neweast Biosciences, Wuhan, China) in which the amino acid sequence is DHYICAKKGGTCNFSPCPLFNRIEGTCYSGKAKCCIR. The mouse macrophage cell line RAW 264.7 was obtained from the American Type Culture Collection (ATCC^®^ TIB-71™, Manassas, VA, USA), and the cells were cultured in RPMI-1640 (# SH30809.01, Hyclone^®^, Logan, UT, USA) containing 10% heat-inactivated FBS (# 10099-141C, Gibco^®^, Grand Island, NY, USA) and 1% penicillin/streptomycin (# SV30010, Hyclone^®^, Logan, UT, USA) at 37°C with 5% CO_2_. The *Salmonella* Typhimurium 14028 strain used in this study was obtained from the American Type Culture Collection (ATCC^®^ 14028™, Manassas, VA, USA), and the bacterial cells were cultured on tryptic soy agar (TSA, # 236950, BD^®^ Difco™, Sparks, MD, USA) or in tryptic soy broth (TSB, # 211825, BD^®^ Difco™, Sparks, MD, USA) at 37°C. Lipopolysaccharides (LPS) from *Escherichia coli* O55:B5 were purchased from Sigma (# L2880, Sigma-Aldrich^®^, MO, USA).

### Physiological Evaluation of the PBD-2 TG Mice

All of the animal experiments were approved by the Animal Experimental Committee and the Transgenic Safety Administrative Committee of Huazhong Agriculture University in accordance with the Hubei Province Laboratory Animal Management Regulations. The PBD-2-expressing TG mice were generated in our previous study (24). In brief, the PBD-2-expressing cassette was released from pcCAG-PBD-2 constructed with AccI used to generate PBD-2-overexpressing TG pigs and composed of the CAG promoter, PBD-2 cDNA and the poly (A) signal of bovine growth hormone (16). TG founder mice expressing PBD-2 were generated by microinjecting a linear PBD-2 expression cassette. PCR was performed to identify the TG mice from the offspring by using the primers NP-f (5-GCTGGTTGTTGTGCTGTCTC-3) and NP-r (5-AGGTCCCTTCAATCCTGTTG-3) for the transgene cassette. RT-PCR was conducted to confirm the expression of PBD-2 in the organs of the TG mice by using the primers PBD-2-f2 (5-ATGAGGGCCCTCTGCTTG-3) and PBD-2-r2 (5-CGGGCAGGGGGAGAAG-3) that targeted PBD-2. The housekeeping gene GAPDH was used as the control with the primers GAPDH-01 (CAATGTGTCCGTCGTGGATCT) and GAPDH-02 (GTCCTCAGTGTAGCCCAAGATG). After the mice were euthanized, blood samples of TG and WT mice were collected. Alanine aminotransferase, aspartate aminotransferase, albumin, globin, albumin/globin, total bilirubin, direct bilirubin, urea, creatinine, urea/creatinine, glucose, potassium, and sodium were analyzed using a blood routine analyser (Mindray, Shenzhen China). Their heart, liver, spleen, lung, kidney, brain and intestinal segments were harvested. The histological morphologies of the mice organs and the body and organ weights of male and female mice were characterized. The intestinal segments were harvested for immunohistochemical analysis. The intestinal tissues were fixed in 10% neutral buffered formalin, dehydrated in a series of alcohols and embedded in paraffin wax. The tissues were sectioned on a sliding microtome at 4 μm. The PBD-2 expression was confirmed by using a PBD-2 monoclonal antibody (IgG1) prepared in our previous study (24) and the Vector M.O.M. Immunodetection Kits (# FMK-2201, Vector Laboratories^®^, Burlingame, CA, USA) which were designed specifically to recognize the mouse primary antibodies on mouse tissues. PBS (# SH30256.01, HyClone^®^, Logan, UT, USA) was used instead of the PBD-2 monoclonal antibody as a negative control.

### Bacterial Infection Assay in Mice

The designs of mouse infection assays are shown in Table S1. For the fatality rate assay, 10 WT male mice and 10 TG mice at the age of 6–8 weeks were orally administered with 5 × 10^8^ CFU of *S*. Typhimurium as previously described (25). The fatality rate of the mice in each group was recorded 15 days after infection.

Four infection experiments were conducted to determine the resistance to *S*. Typhimurium of the TG mice. For body weight measurements, 7 WT and 7 TG mice were orally administered with 2.5 × 10^8^ CFU of *S*. Typhimurium, and the body weight of each mouse was measured on days 1, 2, 3, 4, and 5 after infection. For the detection of the tissue weight ratios, bacterial loads in tissues and tissue lesions after *S*. Typhimurium infection, 9 WT and 8 TG mice were orally administered with 2.5 × 10^8^ CFU of *S*. Typhimurium. The spleen, caecum and body weights of the mice were weighed at 24 h post-infection. The bacterial loads in the tissues were also measured (26). The spleen and caecum were weighed and homogenized in cold PBS at a ratio of 4 ml per gram of tissue and coated plates. The bacterial numbers in the homogenates were counted by serially diluting the homogenates and spreading on plates. Caeca were harvested for histopathological analysis by using the methods described above. The intestinal damage, including epithelial damage, submucosal oedema, and neutrophil aggregation, was observed.

For cytokine detections in WT and TG mice infected with *S*. Typhimurium, 6 WT and 6 TG mice were orally administered with 2.5 × 10^8^ CFU of *S*. Typhimurium. At 24 h post-infection, blood samples of the mice were collected to detect cytokines IL-6 (# EMC004.96), tumor necrosis factor-α (TNF-α, # EMC102a.96), IL-1β (# EMC001b.96), and IL-12 (# EMC006.96) using ELISA kits (Neo-bioscience Technology Company, Shenzhen China). For cytokine detections in the WT mice with or without treatment of PBD-2, the blood samples were collected from the WT mice orally administered with solely 2.5 × 10^8^ CFU of *S*. Typhimurium (*n* = 6) or together with PBD-2 at 5 mg/kg body weight (*n* = 6).

### Detection of Cytokine Levels After the Administration of LPS on Mice by ELISA

The methods of cytokine detections after LPS treatment on mice are shown in Table S1. Six TG mice and six WT mice were separately injected with LPS (16 mg/kg body weight) intraperitoneally. The same number of TG mice and WT mice was injected with 100 μl of sterile PBS as a negative control. The sera from the mice in each group were collected 1 h after the injection to detect cytokines by ELISA.

The WT mice were injected with LPS alone or LPS with PBD-2 (0.5 mg/kg body weight) intraperitoneally (*n* = 6 each group) to detect the effect of synthetic PBD-2 on inflammation induced by LPS. The sera from the mice in each group were collected 1 h after the injection to detect the cytokines by ELISA.

### Bactericidal Activity Assay

*S*. Typhimurium cells were cultured to mid-log phase (OD_600nm_ = 0.7) in TSB. The cultures were centrifuged for 10 min at 1,000 × g, and the cell pellets were diluted in assay medium (10 mM phosphate, pH 7.0, with 1/100 TSB). The antimicrobial activities of PBD-2, TG serum and tissue homogenates were determined by colony counting assay. The mice serum, caecum, colon, or duodenum homogenates were heat inactivated at 56°C for 20 min to exclude the possible effect of complement in tissues. As a control, PBD-2 solution in PBS (50 μg/ml) was also heat-treated at 56°C for 20 min. Afterwards, 5000 CFUs of *S*. Typhimurium were mixed with 100 μl of each sample in a centrifuge tube at 37°C for 1 h. After incubation, 10 serial dilutions from 10 to 1,000-fold in TSB were conducted, and the diluted bacterial cultures were transferred onto TSA plates and incubated at 37°C for 24 h. The numbers of bacterial CFUs were recorded.

### Detection of Cytotoxicity by MTT Assay

3-(4,5-Dimethylthiazol-2-yl)-2,5-diphenyltetrazolium bromide (MTT, # C0009, Beyotime^®^, Shanghai, China) was used to detect the cytotoxicity of PBD-2 by determining cell survivability. Exogenous MTT is reduced into blue and purple crystal formazan by succinate dehydrogenase in the mitochondria of living cells. This product could be dissolved in dimethylsulfoxide (DMSO) and quantitatively determined by detecting the absorbance at 490 nm. In the MTT assay, RAW 264.7 cells were inoculated in 96-well plates and treated with different concentrations of PBD-2 (0, 1, 5, and 10 μg/ml) alone or PBD-2 (0, 1, 5, and 10 μg/ml) with LPS (1 μg/ml). After 16 h, the cells were washed with a fresh medium, added with an MTT solution (20 μl at 5 mg/ml) into each well and incubated at 37°C for 4 h. After the MTT solution was removed, 150 μl of DMSO was added. The absorbance at 490 nm of the 96-well plates was detected using a microplate spectrophotometer (Tecan, Groedig, Austria).

### Construction of PBD-2 Expressing RAW 264.7

The full-length cDNA encoding the PBD-2 peptide (216 bp) was prepared from the liver of a healthy Large White pig through RT-PCR with the primers PBD-2-f1 (5-CCG GAA TTC ATG TGG GCCCTC TGC TTG-3) and PBD-2-r1 (5-CCG CTC GAG TCA GGG TCAGCG GAT GCA-3). The PCR product was cloned into the mammalian expression vector pCAG (# 13461, Addgene^®^, Cambridge, MA, USA) to generate pCAG-PBD-2. The SspI-XhoI fragment containing the CAG promoter (including cytomegalovirus immediate–early enhancer, chicken beta-actin promoter, chicken beta-actin intron and rabbit beta-globin intron) and PBD-2 cDNA, was cut from pCAG-PBD-2 and then inserted between the same restriction sites as those of the mammalian expression vector pcDNA3.1(+) (# V79020, Thermo Fischer Scientific^®^, Waltham, MA, USA). The resulting recombinant plasmid was termed pcCAG-PBD-2. The pcCAG-PBD-2 and empty vector pcDNA3.1(+) were transfected into mouse macrophage RAW 264.7 cells by using Lipofectamine 3000 (# L3000-015, Invitrogen, Thermo Fischer Scientific^®^, Waltham, MA, USA) in accordance with the manufacturer's instructions. The cells were selected with G418 (600 μg/ml) for 14 days, and single cells were isolated to generate stable cell lines that expressed PBD-2. The PBD-2 expression was confirmed through an indirect immunofluorescence assay (IFA) with the PBD-2 monoclonal antibody prepared in our laboratory (24). The expression was further confirmed through qRT-PCR by using the primers PBD-2-f2 and PBD-2-r2.

### Detection of the Cytokine Levels of RAW 264.7 After Treatment With *S*. Typhimurium or LPS

RAW 264.7 cells and RAW 264.7 cells stably expressing PBD-2 were pre-treated with *S*. Typhimurium at MOI = 10:1 for 1 h or with LPS (1 μg/ml) for 16 h, washed with PBS twice and incubated with RPMI 1640 that contained PBS or PBD-2 (10 μg/mL) for 16 h. RAW 264.7 cells stably expressing PBD-2 were maintained in RPMI 1640 that contained PBS for 16 h (*n* = 6 for each group). The expression levels of proinflammatory cytokines in the cell culture supernatant were determined through ELISA in accordance with the manufacturer's instruction.

In the inhibition assay, RAW 264.7 cells were pre-treated with 4 μg/ml NF-κB inhibitor (Celastrol, # ant-cls, InvivoGen^®^, San Diego, CA, USA) or 1 μg/ml TLR4 inhibitor (CLI-095, # tlrl-cli95, InvivoGen^®^, San Diego, CA, USA) for 1 h. Subsequently, RAW 264.7 cells were treated with LPS (1 μg/ml) alone or together with PBD-2 (10 μg/mL) for 16 h (*n* = 6 each group). RAW 264.7 cells treated with PBS were used as control. The levels of the cytokines in the supernatant of the cells were detected through ELISA.

### Detection of Inflammatory Proteins by Western Blot

RAW 264.7 cells were treated with LPS (1 μg/ml) alone or together with PBD-2 (10 μg/mL). After 6 h, the cells were lysed, and inflammatory proteins were detected through Western blot. Proteins were firstly analyzed with SDS-PAGE and then electro-transferred onto a PVDF (#ISEQ00010, Biosharp^®^, Beijing, China) membrane. The PVDF membrane was blocked with 1/100 bovine serum albumin (BSA, #V900933-100G, VETEC, Sigma-Aldrich^®^, St. Louis, MO, USA) at 10 μg/ml at 37°C for 2 h, washed with PBST five times, incubated with the antisera against p-p65 (# 3033), p65 (# 4764), p-ERK (# 4370), ERK (# 4695), p-p38 (# 4511), p38 (# 8690), p-JNK (# 4668), JNK (# 9258) (Cell Signaling Technology^®^, Danvers, MA, USA), and β-actin (# AA128, Beyotime^®^, Shanghai, China) at 2 μg/ml at 37°C for 2 h and washed five times again. The PVDF membrane was subsequently incubated with 1 μg/ml secondary antibody, namely, goat anti-rabbit IgG (# 7074S, Cell Signaling Technology^®^, Danvers, MA, USA), at 37°C for 2 h and washed five times. Lastly, immune-reactive bands were detected using an ECF Western blot system (Tanon 5200 Multi, Shanghai, China) and quantified using AlphaImager 2200 analysis software.

### Endotoxin Activity Analysis

The effect of PBD-2 on the endotoxin activity of LPS was detected via a limulus amoebocyte lysate (LAL) assay by using a chromogenic end-point tachypleus amebocyte lysate kit in accordance with the manufacturer's instructions (27). In brief, the endotoxin catalyzed the activation of proenzyme in LAL, and the activated enzyme catalyzed the substrate to release *p*-nitroaniline (pNA), thereby producing a yellow color. After the termination reagent was used to stop the reaction, the free pNA was measured through spectrophotometry at 405–410 nm in the range of 0.1–1.0 EU/ml. In the assay, PBD-2 at different concentrations (0, 1, 5, and 10 μg/ml) was mixed with LPS at 37°C for 10 min, and the endotoxin activity was evaluated.

### Detection of the Promoter Activity of NF-κB

A luciferase reporter assay was used to detect the promoter activity of NF-κB. RAW 264.7 cells were seeded in a 24-well plate for 24 h and then cotransfected with 800 ng/well of the NF-κB reporter plasmid (pGL3-Basic) and 20 ng/well of pLR-TK. WT marine luciferase (Rluc) served as the reporter gene control vector. After 24 h, the cells were treated with LPS (1 μg/ml) alone or together with PBD-2 (10 μg/mL) for 24 h (*n* = 6 each group), or the cells were pre-treated with PBD-2 at different concentrations (10, 30, and 60 μg/ml) for 30, 60, and 120 min. In the inhibition assay, the cells were pre-treated with 1 μg/ml TLR4 inhibitor for 1 h and treated with LPS (1 μg/ml) alone or together with PBD-2 (10 μg/mL) for 24 h (*n* = 6 each group). Luciferase units were detected to show the promoter activity of NF-κB (28).

### Determination of the mRNA Level of NF-κB

The cDNA of the RAW 264.7 cells treated with LPS (1 μg/ml) alone or together with PBD-2 (10 μg/mL) for 4 h (*n* = 6 each group) was used as a template by using the primers NF-κB-01 (AACAAAATGCCCCACGGTTA) and NF-κB-02 (GGGACGATGCAATGGACTGT) and the primers GAPDH-01 and GAPDH-02 for the housekeeping gene GAPDH to detect the expression level of NF-κB. The relative expression level of NF-κB is shown as 2^−Δ*Ct*^ (mean ± SEM), where ΔCt = Ct _NF−κ*B*_−*Ct*_GAPDH_. The protocol consisted of 40 replication cycles. The Ct values above 34 cycles were considered non-detectable.

### Detection of Interactions Between PBD-2 and LPS or TLR4 Through Isothermal Titration Calorimetry (ITC)

The interactions between PBD-2 and LPS or TLR were determined using a Nano-ITC machine (TA Instruments Limited, New Castle, DE, USA). The ITC assay was performed at 25°C with PBD-2 in the syringe (concentration = 0.75 mM) and LPS in the calorimeter cell (concentration ranging from 0.010 to 0.050 mM). As a control, PBD-2 (concentration = 0.75 mM) was loaded into the syringe, and the PBS was loaded into the calorimeter cell. The ITC assay was performed at 25°C with PBD-2 in the syringe (concentration = 0.215 mM) and TLR4 (# RPA753Mu01, Cloud-clone^®^, Wuhan, China) in the calorimeter cell (concentration ranging from 0.016 to 0.050 mM) to detect the PBD-2–TLR4 interaction. As a control, PBD-2 was loaded into the syringe (concentration = 0.215 mM), and PBS was loaded into the calorimeter cell.

### Detection of Interactions Between PBD-2 and TLR4 by Far-Western Blot

TLR4 and BSA proteins as a control were subject to SDS-PAGE and electro-transferred onto a PVDF membrane. The PVDF membrane was blocked with 10 μg/ml BSA in PBST at 37°C for 2 h, washed five times with PBST and incubated with PBST that contained 10 μg/ml PBD-2 or 10 μg/ml BSA as a control at 37°C for 2 h. After the membranes were washed, the PVDF membrane was incubated with anti-PBD-2 antisera (with a titer 1:500,000) at 37°C for 2 h and washed five times with PBST. The membrane was then incubated with the secondary antibodies, namely, goat anti-mouse IgG (# AS003, ABclonal^®^, Wuhan, China), at 37°C for 2 h and washed five times with PBST. Lastly, immune-reactive bands were detected using an ECF Western blot system (Tanon 5200 Multi, Shanghai, China).

### Detection of Interaction Between PBD-2 and TLR4 Through Coimmunoprecipitation (Co-IP)

A Co-IP assay was conducted using a Co-IP kit (# abs955, Absin^®^, Shanghai, China) in accordance with the manufacturer's instructions. In brief, RAW 264.7 cells were cultured in dishes of 10 cm in diameter. The cells (1 × 10^7^) were treated with different concentrations of PBD-2 (1, 10, 30, and 50 μg/ml) for 16 h and lysed using a lysis buffer that contained protease inhibitors for 16 h. The soluble faction of the cell lysate was collected after centrifugation at 14,000 × g at 4°C for 10 min. Afterwards, 2 μl of PBD-2 antisera (with titer 1:500,000) or 2 μl of mouse IgG antisera (# sc-2025, Santa Cruz^®^, CA, USA) as control were incubated with 200 μg of the cell lysate at 4°C for 12 h. Protein A/G beads (5 μl) were added and incubated at 4°C for 12 h. The precipitates with the immune complexes were collected by centrifugation at 12,000 × g at 4°C for 1 min. The beads were washed with a wash buffer. TLR4 in the precipitates was detected with Western blot by using an antibody against TLR4 (# sc-293072, Santa Cruz^®^, Santa Cruz, CA, USA).

### Statistical Analysis

Statistical analysis was performed using Student's two-tailed *t*-test with GraphPad (GraphPad^®^, La Jolla, CA, USA), and the survival rates of the mice were compared using a log-rank test within GraphPad. Data were displayed as means ± SEM. Values with *p* < 0.05 were considered significantly different (^*^p < 0.05; ^**^p < 0.01; ^***^p < 0.001; ^****^p < 0.0001).

## Results

### Physiological Characterization of PBD-2 TG Mice

**Figure 1:**
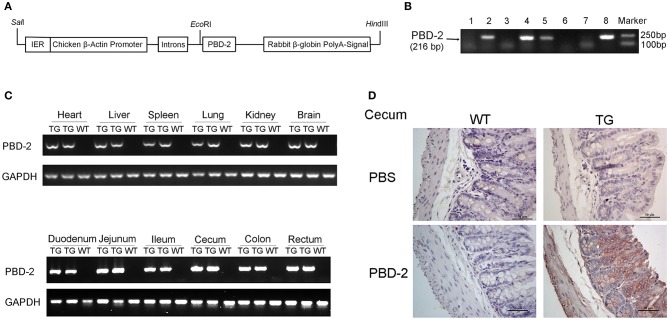
Physiological characterization of PBD-2 TG mice. **(A)** PBD-2 expression box used to generate PBD-2-expressing TG mice. **(B)** PCR detection of TG mice. **(C)** Detection of the expression of PBD-2 in the organs of TG mice by qRT-PCR. **(D)** Detection of the expression of PBD-2 in caecum by immune histochemical analysis. The locations with brown colors show positive signals that indicate PBD-2 expression.

F0 TG mice expressing PBD-2 were generated by microinjection to assess the antibacterial potential of PBD-2 *in vivo*. In these TG mice, the expression of *pbd-2* was driven by the CAG promoter (Figure 1A). PCR was performed using the genomic DNA from the offspring of the F0 TG mice, and the results showed the successful expression of *pbd-2* (Figure 1B). The RT-PCR results revealed that PBD-2 was expressed in all of the organs of the TG mice (Figure 1C). The immunohistochemistry staining of the intestinal segments by using PBD-2 monoclonal antibody showed strong PBD-2 signals (brown). The caecum is shown in Figure 1D, and the other intestinal sections are illustrated in Figure S1A. These findings verified the expression of PBD-2 in the TG mice. The TG mice did not significantly differ from the WT mice in terms of body weight (Figure S1B) and the ratio of organ weight to total body weight (Figure S1C). The physiological and biochemical indexes of the blood sample from the TG mice also displayed no difference compared with those of the WT mice (Table S2). Therefore, the expression of *pbd-2* did not significantly affect the physiological features of mice.

### PBD-2 Overexpression Protected the Mice From *S*. Typhimurium Infection

**Figure 2:**
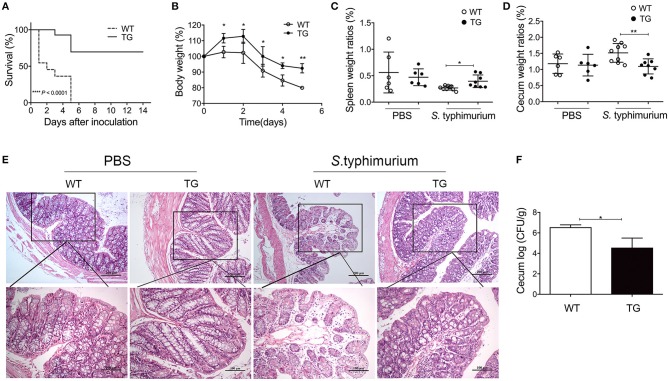
PBD-2 overexpression protected mice from *S*. Typhimurium infection. **(A)** Survival rates of WT and TG mice after infection of *S*. Typhimurium. WT and TG mice were orally infected with 5 × 10^8^ CFU of *S*. Typhimurium (*n* = 10). The survival numbers of mice were recorded daily. **(B–F)** WT and TG mice were orally infected with *S*. Typhimurium at 2.5 × 10^8^ CFU (*n* ≥ 6). **(B)** Comparison of percentages of body weight loss (currently body weight/original body weight before infection) between TG mice and WT mice daily (*n* = 7 in each group). **(C)** Comparison of spleen weight ratios (organ weight/body weight) between TG and WT mice at 24 h post-infection (WT, *n* = 9; TG, *n* = 8). **(D)** Comparison of caecum weight ratios (organ weight/body weight) between TG and WT mice at 24 h post-infection (WT, *n* = 9; TG, *n* = 8). **(E)** Comparison of pathological changes of caecum between TG and WT mice at 24 h post-infection. Results are shown as the histological sections of ceca after H&E staining. Upper panel, magnification = 100-fold; lower panel, magnification = 200-fold. **(F)** Comparison of bacterial loads in the ceca between TG and WT mice at 24 h post-infection (*n* = 6). Mice treated with PBS were used as negative control (*n* = 6). Data are represented as means ± SD. *p < 0.05, **p < 0.01.

A mouse infection model was used to explore the possible role of PBD-2 in protecting mice against *S*. Typhimurium infection. Figure 2A shows that 70% of the TG mice survived after inoculation with 5 × 10^8^ CFU of *S*. Typhimurium, whereas none of the WT mice survived (*p* < 0.0001). An infection experiment was performed with a relatively lower bacterial dose (2.5 × 10^8^ CFU) to determine the influence of PBD-2 on colitis caused by *S*. Typhimurium. Under this condition, all the WT and TG mice survived until the end of the experiments. The TG mice suffered from a less degree of body weight loss from day 2 onwards (*p* < 0.05) (Figure 2B). By contrast, the WT mice exhibited more severe splenic atrophy and caecal enlargement compared with the TG mice on day 2 post-infection (*p* < 0.05) (Figures 2C,D). Pathological changes in the caecum were assessed by histological examination to determine the degree of tissue lesion. Figure 2E demonstrates that the caecum of the TG mice showed significantly milder intestinal damage (epithelial damage and submucosal oedema) and less neutrophil aggregation compared with that of the WT mice. The TG mice also had a smaller amount of bacterial load in the caecum compared with the WT mice (*p* < 0.05) (Figure 2F). Collectively, the PBD-2 TG mice represented an increased resistance to *S*. Typhimurium infection compared with that of the WT mice.

### Bactericidal Activity of PBD-2 Against *S*. Typhimurium

**Figure 3:**
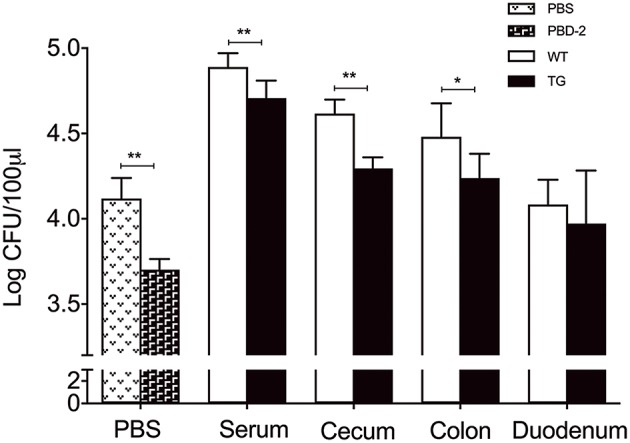
Bactericidal activity of PBD-2 against *S*. Typhimurium. Synthetic PBD-2 after heat treatment and bacteria of 5000 CFUs were incubated in 10 mM phosphate with 1/100 TSB for 1 h. Bacteria were also incubated for 1 h with heat-inactivated serum, homogenates of caecum, colon and duodenum of TG and WT mice. The bactericidal activity of PBD-2 is shown as the survived bacterial numbers after incubation with PBD-2 under the same heat-treatment or serum/tissue homogenates compared with the negative control. Data are represented as means ± SD (WT, *n* = 6; TG, *n* = 6). *p < 0.05, **p < 0.01.

The antibacterial effects of the serum and the tissue homogenates of the mice were tested to investigate why the TG mice were more resistant to *S*. Typhimurium infection than the WT ones. After 1 h of *S*. Typhimurium incubation with heat-inactivated serum and homogenates of caecum and colons of TG and WT mice, respectively, the number of the survived bacteria in the serum and tissue homogenates of the WT mice was significantly higher than that in the serum and tissue homogenates of the TG mice (*p* < 0.05) (Figure 3). The *in vitro* assay also showed that the synthetic PBD-2 after the same heat treatment as the control elicited a bacterial killing effect against *S*. Typhimurium (Figure 3). Our data suggested that PBD-2 had a direct bactericidal activity against *S*. Typhimurium *in vivo* and *in vitro*.

### PBD-2 Could Alleviate *S*. Typhimuriumi- and LPS-Induced Inflammation

**Figure 4:**
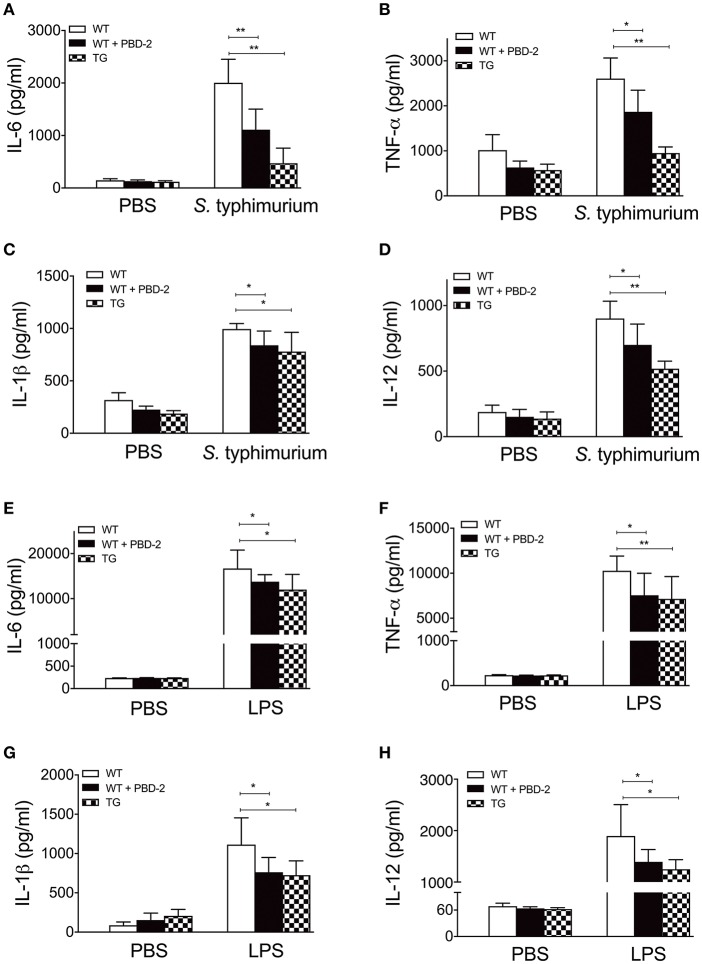
PBD-2 could alleviate *S*. Typhimuriumi- and LPS-induced inflammation. TG and WT mice were orally administered with 2.5 × 10 ^8^ CFU of *S*. Typhimurium. In another group, WT mice were orally administered with 2.5 × 10 ^8^ CFU of *S*. Typhimurium together with PBD-2. At 24 h post-infection, blood samples of the mice were collected for detection of cytokines **(A–D)**. In another experiment, WT mice, TG mice, and WT mice treated with PBD-2 were injected with LPS (16 μg/g). The blood samples were collected from the mice 1 h after the injection to detect cytokines **(E–H)**. Mice treated with PBS were used as negative control. The proinflammatory mediators TNF-α, IL-6, IL-1β, and IL-12 in the mice serum were examined by ELISA. Data are represented as means ± SD (WT, *n* = 6; TG, *n* = 6). *p < 0.05, **p < 0.01.

We subsequently measured the production of proinflammatory mediators (26, 29) in the serum of *S*. Typhimurium infected WT and TG mice. The levels of TNF-α, IL-6, IL-1β, and IL-12 in the serum of the TG mice were significantly lower than those of the WT mice (*p* < 0.05) (Figures 4A–D). In another group, the WT mice were orally administered with synthetic PBD-2 and *S*. Typhimurium. Similarly, the mice treated with PBD-2 and *S*. Typhimurium secreted significantly lower levels of proinflammatory cytokines than the mice infected with *S*. Typhimurium alone did (*p* < 0.05) (Figures 4A–D). The LPS-induced inflammatory response was also detected in the TG and WT mice. The serum from the WT mice injected with LPS showed significantly increased levels of proinflammatory cytokines compared with that from the mice treated with PBS. The LPS-induced production of cytokines was significantly lower in the TG mice than in the WT mice (*p* < 0.05) (Figures 4E–H), thereby indicating the suppressing effect of PBD-2 on cytokine production. The WT mice injected with synthetic PBD-2 and LPS had significantly lower levels of secreted TNF-α, IL-6, IL-1β, and IL-12 than those injected with LPS alone (*p* < 0.05) (Figures 4E–H). These data Taken together, these data indicated that PBD-2 reduced *S*. Typhimurium- or LPS-induced inflammatory responses in mice.

### PBD-2 Inhibited LPS-Induced Inflammation via the NF-κB Signaling Pathway

**Figure 5:**
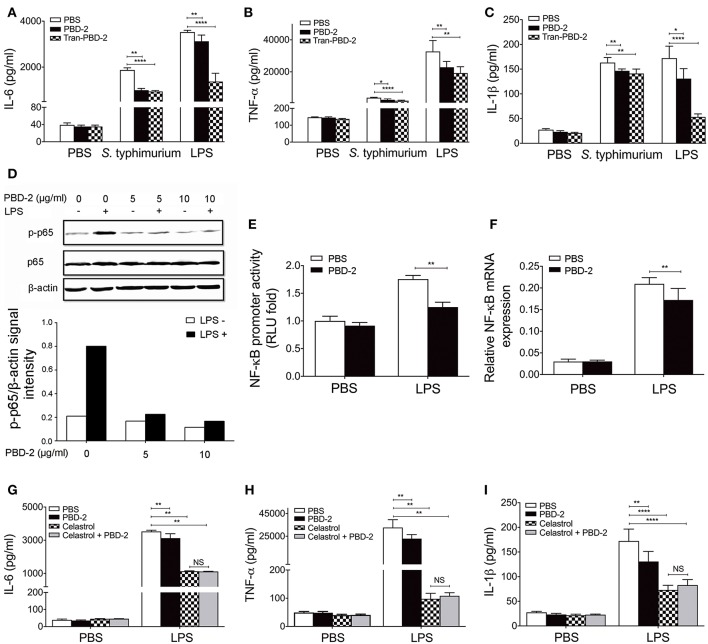
PBD-2 inhibited LPS-induced inflammation *via* NF-κB signaling pathway. **(A–C)** Levels of proinflammatory cytokines in the supernatants of RAW 264.7, RAW 264.7 supplemented with PBD-2 or RAW 264.7 expressing PBD-2 (Tans-PBD-2 RAW 264.7) after treatment with *S*. Typhimurium or the stimuli LPS. Cells treated with PBS were used as negative control. **(D)** Cells were treated with LPS with or without various concentrations of PBD-2, then expression of p65 and p-p65 was examined by Western blot (upper). The signal intensity of each band was quantified (lower). **(E)** Cells were incubated with LPS alone or with PBD-2. The NF-κB promoter activity was detected by luciferase reporter assay. Results are presented as fold induction of luciferase compared with negative control. Cells treated with PBS were used as negative control. **(F)** Cells were incubated with LPS alone or with PBD-2, and the mRNA levels of NF-κB were detected by RT-PCR. **(G–I)** RAW 264.7 cells were pre-treated with NF-κB -inhibitor (Celastrol), then the cells were treated with LPS alone or together with PBD-2. Cells treated with PBS were used as negative control. Levels of proinflammatory cytokines in the supernatants of the cells were detected. All data are represented as means ± SD (*n* = 6 each group). NS, *p* > 0.05, *p < 0.05, **p < 0.01, ****p < 0.0001.

MTT assay was performed to confirm whether different concentrations of PBD-2 could affect the viability of RAW 264.7 cells (Figure S2). A PBD-2-expressing RAW 264.7 cell line was generated to understand the immune response signaling pathway involving PBD-2. The expression of PBD-2 in RAW 264.7–PBD-2 was confirmed by indirect immunofluorescence (Figure S3A) and RT-PCR (Figure S3B). RAW 264.7 cells were then treated with *S*. Typhimurium alone or with synthetic PBD-2, and the secretion levels of cytokines were measured. PBD-2 significantly inhibited the secretion of TNF-α, IL-6, and IL-1β after *S*. Typhimurium treatment (*p* < 0.05) (Figures 5A–C). The levels of TNF-α, IL-6, and IL-1β were significantly lower in the RAW 264.7–PBD-2 cells than in the RAW 264.7 cells after *S*. Typhimurium treatment (*p* < 0.01) (Figures 5A–C). Afterwards, RAW 264.7 cells were treated with LPS alone or together with synthetic PBD-2, and the secretion of cytokines was detected. The TNF-α, IL-6, or IL-1β level was significantly reduced in RAW 264.7 cells supplemented with PBD-2 or in the RAW 264.7–PBD-2 cells (*p* < 0.05) (Figures 5A–C).

Furthermore, PBD-2 was found to suppress the LPS-induced NF-κB p65 phosphorylation (Figure 5D) but did not affect the LPS-induced phosphorylation of ERK, p38, and JNK (Figure S4). PBD-2 also inhibited the promoter activities of NF-κB after LPS induction (*p* < 0.01) (Figure 5E). Consistently, PBD-2 could reduce the mRNA levels of NF-κB after LPS induction (*p* < 0.01) (Figure 5F). The RAW 264.7 cells were pre-treated with the NF-κB inhibitor and then treated with LPS alone or with synthetic PBD-2, and the secretion of cytokines was measured to ensure that PBD-2 could inhibit the LPS-induced inflammation *via* the NF-κB pathway. The NF-κB inhibitor significantly reduced the secretion of TNF-α, IL-6, and IL-1β triggered by LPS, whereas PBD-2 did not affect the secretion of these cytokines in the presence of NF-κB inhibitor (NS, *p* > 0.05) (Figures 5G–I). Our data showed that PBD-2 could inhibit the LPS-induced inflammation cytokines by suppressing the NF-κB pathway.

### PBD-2 Could Directly Interact With TLR4

**Figure 6:**
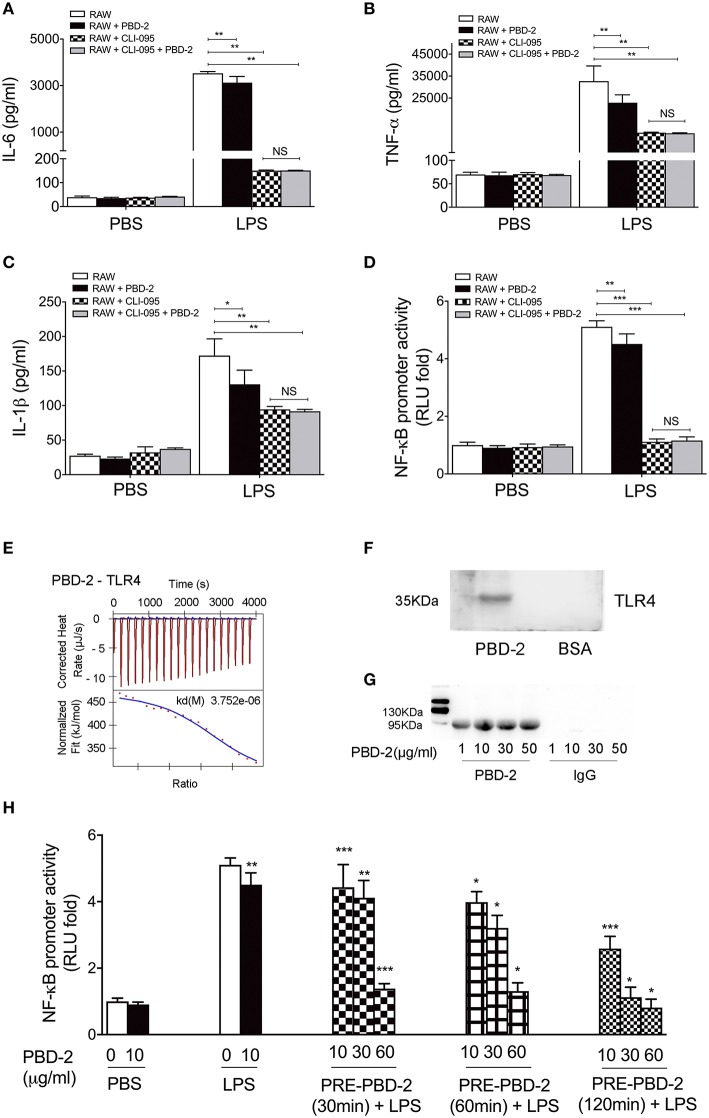
PBD-2 could directly interact with TLR4. **(A–C)** RAW 264.7 cells were pre-treated with TLR4 inhibitor (CLI-095), then the cells were treated with LPS alone or together with PBD-2. Cells treated with PBS were used as negative control. Levels of proinflammatory cytokines in the supernatants of the cells were detected. **(D)** RAW 264.7 cells were pre-treated with TLR4 inhibitor (CLI-095) and then incubated with LPS alone or with PBD-2, and the NF-κB promoter activity was detected by luciferase reporter assay. Cells treated with PBS were used as negative control. **(E)** Interaction of PBD-2 with TLR4 detected by ITC. PBS was used as negative control. **(F)** Interaction of PBD-2 with TLR4 detected by far-Western blot. **(G)** Interaction of PBD-2 with TLR4 detected by Co-IP on RAW 264.7 cells. **(H)** Detection of NF-κB promoter activity after pre-incubation of PBD-2 with RAW 264.7 cells. Cells were pre-incubated with PBD-2 at 10, 30, or 60 μg for 30, 60, or 120 min (PRE-PBD-2), then LPS was supplemented and incubated with the cells. LPS was added at the same time with PBD-2 or alone as control. PBS was added instead of LPS as negative control. NF-κB promoter activity was determined by luciferase reporter assay. Data are represented as means ± SD (*n* = 6 each group). *p < 0.05, **p < 0.01, ***p < 0.001.

RAW 264.7 cells were pre-treated with TLR4 inhibitor and then treated with LPS alone or with synthetic PBD-2. The secretion of cytokines was measured to detect how PBD-2 interfere with the NF-κB pathway after LPS stimulation. The TLR4-inhibitor significantly reduced the secretion of TNF-α, IL-6, and IL-1β triggered by LPS, whereas PBD-2 did not affect the secretion of these cytokines in the presence of TLR4 inhibitor (NS, *p* > 0.05) (Figures 6A–C). The TLR4 inhibitor also inhibited the LPS-induced promoter activity of NF-κB in RAW 264.7, whereas PBD-2 did not influence the NF-κB promoter activity in the presence of TLR4 inhibitor (NS, *p* > 0.05) (Figure 6D). The effects of PBD-2 on the endotoxic activity of LPS and the interactions of PBD-2 with LPS or TLR4 were tested. The results demonstrated that different concentrations of PBD-2 did not affect the endotoxic activity of LPS (Figure S5). Interaction studies showed that PBD-2 did not bind to LPS (Figure S6). However, a direct interaction between PBD-2 and TLR4 was observed (Figure 6). ITC was used *in vitro* to show that the synthetic PBD-2 and the purified TLR4 could interact with each other with a binding affinity of 3.752 μM (Figure 6E). The far-Western blot assay also showed that PBD-2 could bind to TLR4 but not to BSA on the PVDF membrane (Figure 6F). After the RAW 264.7 cells were treated with PBD-2 at different concentrations, the cell lysates captured through immunoprecipitation by using the PBD-2 antisera could react with the antibody against TLR4, whereas the negative control with IgG instead of PBD-2 antisera did not react with the TLR4 antibody (Figure 6G). The cells were treated with different concentrations (10, 30, and 60 μg/ml) of PBD-2 for 30, 60, 120, or 240 min and then treated with LPS to detect the effect of the PBD-2–TLR4 interaction on NF-κB activation after LPS stimulation. The inhibitory effect of PBD-2 on the promoter activity of NF-κB was positively correlated with dose and incubation time with PBD-2 (*p* < 0.05) (Figure 6H). The result indicated that the binding of PBD-2 to TLR4 could be one of the explanations why the NF-κB pathway was inhibited after the cells were treated with LPS.

## Discussion

The antibiotic resistance of pathogenic bacteria has become a global threat (30). Defensins are natural peptides secreted by many species and implicated in resisting bacterial infection (31). PBD-2 is one of the defensins expressed in pigs. Previous studies have revealed that PBD-2 works as an effective antibacterial agent against various bacteria *in vitro* (11). The invasion of *S*. Typhimurium can increase the expression of PBD-2 in porcine epithelial cells (32). PBD-2 can alleviate mucosal damage caused by dextran sodium sulfate (DSS) in mice (33). PBD-2 also improves the growth performance of piglets (15). Our previous study showed that the overexpression of PBD-2 could increase the protection of pigs against bacterial infection (16). In the present study, we aimed to further evaluate the anti-infection roles of PBD-2 and reveal the underlying mechanisms. Mice express four types of beta-defensins (MBD-1,−2,−3, and−4) (34). These MBDs showed low identities with PBD-2 ( ≤ 38%). Therefore, TG mice expressing PBD-2 were constructed to be used as an *in vivo* PBD-2 expression model. According to our results, the TG mice had similar physiological features to the WT mice. However, the TG mice showed significantly enhanced resistance to *S*. Typhimurium infection. The anti-infection effect is due to the direct killing effect of PBD-2 against bacteria because the bactericidal activities of the serum and the homogenate of the tissues of TG mice were higher than those of the WT mice. This observation is consistent with our previous study which demonstrated that TG pigs overexpressing PBD-2 display an enhanced resistance to *A. pleuropneumoniae*, and the bactericidal activity of the serum of TG pigs is higher than that of WT pigs (16). We also confirmed that the synthetic PBD-2 reduced the viability of *S*. Typhimurium *in vitro*. These results agree with a previous study which showed that PBD-2 has a direct bactericidal action *in vitro* (11). Our studies further verified the antibacterial ability of PBD-2 *in vivo*. Porcine beta-defensin 1, another defensin from pigs and homolog of human beta-defensin 2, protects against *Bordetella pertussis* infection in pigs (35). Other defensins, such as β-defensins of human (hBD-1 and hBD-2) (36) and β-defensin 3 (rMBD3) (37) of mice, have antibacterial effects *in vivo*. The main mechanism of AMPs to kill bacteria is the destruction of bacterial cell membranes (38). Some AMPs can enter the cells and bind to intracellular molecules, thereby leading to bacterial death (39). Many AMPs have a broad-spectrum bactericidal activity and do not bind to specific receptors on bacterial surfaces. Consequently, pathogens rarely develop resistance to AMPs (2). Thus, the AMPs, including PBD-2, are promising alternatives to antibiotics. On the other hand, breeding for disease-resistant animals by introducing PBD-2 may also be an effective strategy to combat infectious diseases.

In this study, PBD-2 TG mice did not show a complete resistance to *S*. Typhimurium infection. Our TG pigs overexpressing PBD-2 display an enhanced but incomplete resistance to *A. pleuropneumoniae* (16). These phenomenons might be due to the expression level of PBD-2 *in vivo*. *In vitro*, the antibacterial activity of PBD-2 is positively related to its concentration (11). Although the expression level of PBD-2 in the TG mice can reach 10^4^ ng/g tissue according to our previous study (24), it may be insufficiently high to deal with all the infected bacteria *in vivo*. Another fact is that some virulence factors and metabolites of bacteria can inhibit the expression of defensins (40, 41). Hence, further increasing the expression level of PBD-2 *in vivo*, such as by introducing more robust promoter or more copies of PBD-2, might increase the disease resistance abilities of animals.

The colitis caused by *S*. Typhimurium mainly results from intestinal mucosal inflammation after the breakage of intestinal epithelial barrier by pathogens (26). In this study, the PBD-2 TG mice exhibited a significantly alleviated epithelial damage, submucosal intestinal oedema and less neutrophil accumulation in the caecum tissue, thereby demonstrating reduced inflammatory responses compared with those in the WT mice. This result could be due to the bactericidal activity of PBD-2 expressed in the gut of the TG mice because the bactericidal abilities of the homogenates of the caecum and the colon of the TG mice were higher than those of the WT mice. Another reason for the reduced colitis of the TG mice was the suppressing effect of PBD-2 in inflammation discovered in this study. A previous study confirmed that porcine intestinal cell lines showed a high expression of the proinflammatory cytokines TNF-α and IL-1β after the stimulation of LPS from *S*. Typhimurium infection (42). In the current study, the release of the inflammatory cytokines IL-6, TNF-α, IL-1β, and IL-12 in the serum of the TG mice was reduced compared with that of the WT mice after infection with *S*. Typhimurium or administration with LPS. The WT mice injected with synthetic PBD-2 also displayed suppressed levels of cytokines after stimulation with LPS or *S*. Typhimurium (Figure 4). Thus, PBD-2 could reduce the inflammatory responses caused by *S*. Typhimurium infection and LPS administration. After a macrophage cell line expressing PBD-2 was constructed or the macrophages were treated with PBD-2, the results revealed that PBD-2 inhibited the levels of inflammatory cytokines. The inflammatory responses of the RAW 264.7 cells expressing PBD-2 were lower than those of the cells supplemented with synthetic PBD-2 (Figures 5A–C). PBD-2 expressed by the cells might possess natural spatial conformation and was more stable than synthetic PBD-2. Taken together, PBD-2 could act as an anti-inflammatory factor protecting animals from excessive inflammation. Numerous members of AMPs have been found to be immune regulators. They can recruit and activate immune cells, regulate cytokine levels or inhibit inflammatory responses (31). Human AMPs LL-37, IDR-1, and IDR-1002 have been observed to suppress the release of inflammatory cytokines (5). The human β-defensin 3 (hBD3) has also been found to possess an anti-inflammatory function by suppressing TNF-α and IL-6 secretion (43). In comparison with untransfected cells, intestinal epithelial cells transfected with genes encoding hBD-2 and hBD-3 and infected with *S*. Typhimurium display a decreased expression level of proinflammatory cytokines (44). In another study, PBD-2 attenuates inflammation in DSS-induced colitis which mimics chronic inflammatory bowel disease (33). Therefore, PBD-2, similar to many human AMPs, had a direct bactericidal activity and could control inflammation.

Our study further discovered that PBD-2 could inhibit the LPS-activated NF-κB signaling pathway but not the ERK, p38, and JNK MAPK pathways. This result is consistent with that of a previous study which showed that PBD-2 suppresses the DSS-induced inflammation by suppressing the NF-κB signaling pathway (33). Similarly, hBD3-3 can block NF-κB-dependent inflammation (45). However, rhesus θ-defensin 1 (RTD-1) suppresses inflammatory responses through several signaling pathways, including p38, JNK, ERK2, CREB, and HSP27 (46). AMPs have various structures which may lead to their different actions on signaling pathways in immune cells (10).

The mechanism of how AMPs modulate immune signaling pathways has yet to be fully understood. Evidence shows that this mechanism includes interfering with TLRs on cell surfaces by directly binding to TLRs or neutralizing TLR ligands (47). Our results indicated that PBD-2 itself did not activate NF-κB (Figure 5D) and did not affect cytokine secretion (Figures 5A–C). We also found that PBD-2 neither bound to LPS nor affected the LPS activity. Instead, it interacted with TLR4. Cells pre-incubated with PBD-2 showed a suppressed activity of NF-κB in a time- and dose-dependent manner. Therefore, the binding of PBD-2 to TLR4 may competitively inhibit the LPS-induced activation of the NF-κB signaling pathway, thereby restricting the release of downstream inflammatory cytokines. These results are different from those of previous studies showing that the human AMP LL37 (48) and porcine peptides PR39, PGs, and NK-lysin (49) exert anti-endotoxin abilities by either disrupting LPS or inhibiting the LPS-induced production of TNF-α. In another study, TLR3-dependent immune responses are blocked by LL-37 (6). The inhibitory effect was attributed to the primary binding of the AMP to the TLR3 ligand, namely, viral dsRNA poly (I:C). HBD3 could inhibit the LPS-induced inflammation by repressing the signaling pathways downstream of TLR4, including MyD88-dependent and MyD88-independent pathways. However, hBD3 does not bind to LPS, and the hBD3-induced suppression of immune responses is independent of LPS binding (50). hBD3 can interact with TLR1 and TLR2, leading to the activation of the MyD88-dependent pathway (50). These findings suggested the diverse action modes of AMPs on cell membrane receptors. Other PBD-2 receptors may also exist on the surface of immune cells, and this aspect needs further investigations. PBD-2 binding to TLR4 may affect other intracellular pathways that are downstream of TLR4, such as intrinsic and extrinsic apoptosis pathways (51), chemotactic factor secretion mediated by IRF5 (52) and interferon secretion regulated by IRF3 (51). The possible influence of PBD-2 on these pathways should be further studied.

In summary, in this study, the anti-infection role of PBD-2 was confirmed by using TG mice with *S*. Typhimuriumi infection. PBD-2 displayed a direct bactericidal activity *in vitro* and in the serum and tissues of TG mice. PBD-2 also served as an immune modulator to restrict inflammation after bacterial infection or LPS treatment in mice and macrophage models. Interference with the TLR4/NF-κB pathway contributed to the suppression effect of PBD-2 on the release of inflammatory cytokines. PBD-2 might also interact with other signaling pathways or receptors. However, such interaction should be further studied to fully show the regulatory roles of PBD-2 as an immune modulator.

## Ethics Statement

Animal experiments were approved by the Laboratory Animal Monitoring Committee of Huazhong Agricultural University and performed in strict accordance with the recommendations in the Guide for the Care and Use of Laboratory Animals of Hubei Province, China (53).

## Author Contributions

CH designed and performed the experiments, analyzed the data, and wrote the manuscript. XiY, JH, XL, XiaY, and HJ performed the experiments. QH analyzed the data. LL and RZ conceived the project, analyzed the data, and wrote the manuscript.

### Conflict of Interest Statement

The authors declare that the research was conducted in the absence of any commercial or financial relationships that could be construed as a potential conflict of interest.

## Supplementary Material

Figure S1 **(A)** Detection of the expression of PBD-2 in the duodenum, jejunum, ileum, colon, and rectum through immunohistochemical analysis. The positive signals showing the PBD-2 expression are denoted by a brown. **(B)** Comparison of body weights between TG and WT mice. **(C)** Comparison of weight ratios of organs, including heart, liver, spleen, lung, kidney, and brain (organ weight/body weight) between TG and WT mice. Data are represented as means ± SD (WT, *n* ≥ 6; TG, *n* ≥ 6).

Figure S2 Effect of PBD-2 on the viability of RAW 264.7 cells. Cell proliferation rate was detected by MTT assay. RAW 264.7 cells were incubated with PBD-2 at 1, 5, and 10 μg/ml alone or together with LPS (1 μg/ml). Data are represented as means ± SD (*n* = 6 each group).

Figure S3 PBD-2 expressing RAW 264.7 cells were confirmed through indirect IFA by using the antiserum of PBD-2 **(A)** and qRT-PCR by using the PBD-2 detection primers **(B)**.

Figure S4 Effects of PBD-2 on ERK, p38, and JNK signaling pathways. Cells were treated with LPS for 30 min with or without various concentrations of PBD-2. The expression levels of ERK, p-ERK, p38, p-p38, JNK, p-JNK, and β-actin were examined by Western blot **(A)**. The signal intensity of each band was quantified **(B–D)**.

Figure S5 Effect of PBD-2 on the endotoxin activity. Endotoxin activity was detected via a LAL assay. Cells were treated with LPS (1 μg/ml) with or without various concentrations of PBD-2 (1, 5 and 10 μg/ml). Data are represented as means ± SD (n = 6 each group).

Figure S6 Interaction of PBD-2 with LPS detected by ITC. PBD-2 was placed in a syringe (concentration = 0.75 mM) and LPS was added in the calorimeter cell (concentration = 0.050 mM). PBS was placed in the calorimeter cell as a negative control.

Table S1 Assays of bacterial infection or LPS treatment of mice.

Table S2 Physiological and biochemical indexes of the blood sample from TG and WT mice.
