# Supplementary figures and images for: Porcine Beta-Defensin 2 Provides Protection Against Bacterial Infection by a Direct Bactericidal Activity and Alleviates Inflammation via Interference With the TLR4/NF-κB Pathway
Source: Front Immunol. 2019 Jul 18;10:1673. doi: 10.3389/fimmu.2019.01673 (PMC6657668; doi:10.3389/fimmu.2019.01673)

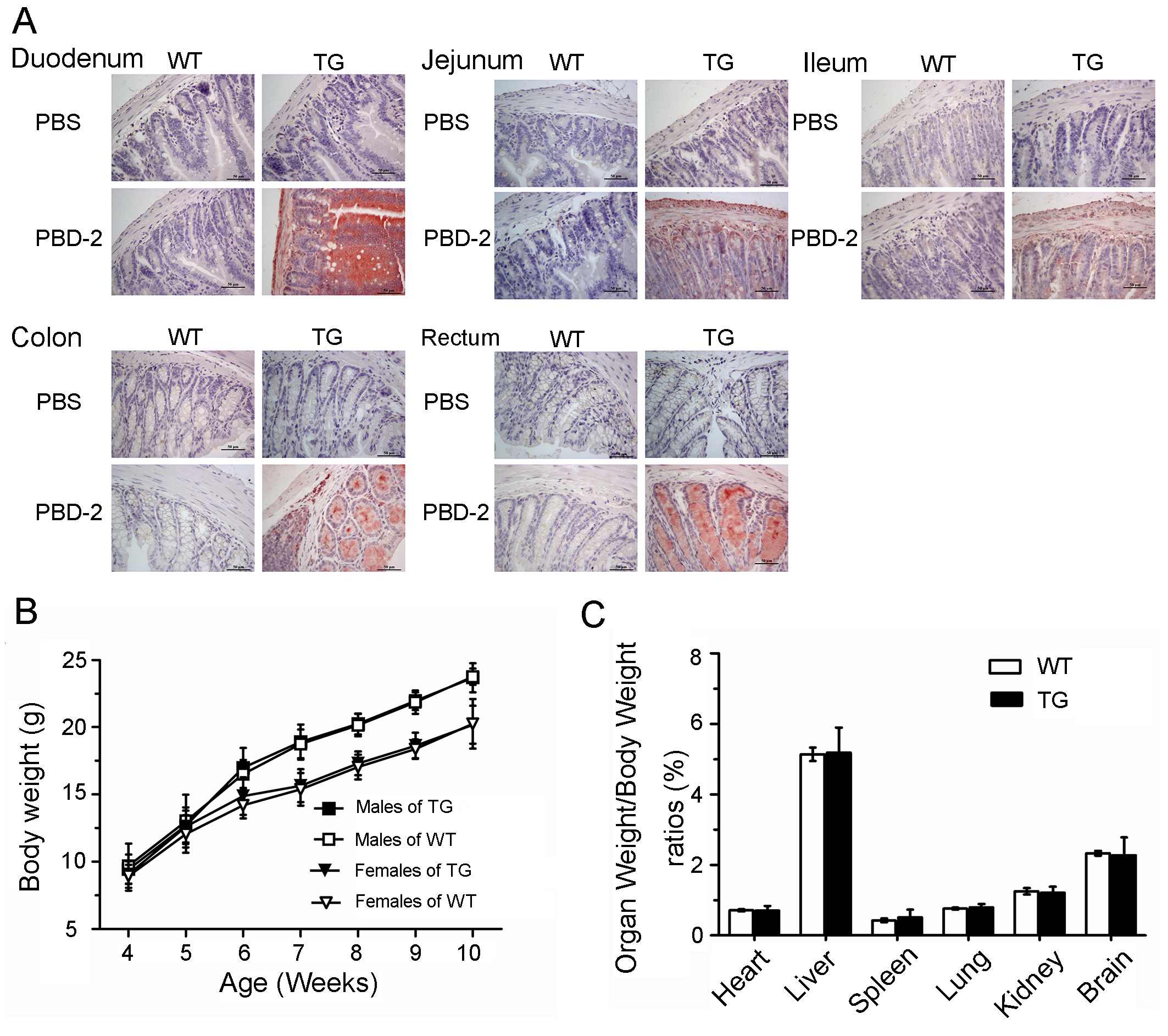

Supplement: Figure S1 — (A) Detection of the expression of PBD-2 in the duodenum, jejunum, ileum, colon, and rectum through immunohistochemical analysis. The positive signals showing the PBD-2 expression are denoted by a brown. (B) Comparison of body weights between TG and WT mice. (C) Comparison of weight ratios of organs, including heart, liver, spleen, lung, kidney, and brain (organ weight/body weight) between TG and WT mice. Data are represented as means ± SD (WT, n ≥ 6; TG, n ≥ 6). [file Image_1.TIF]

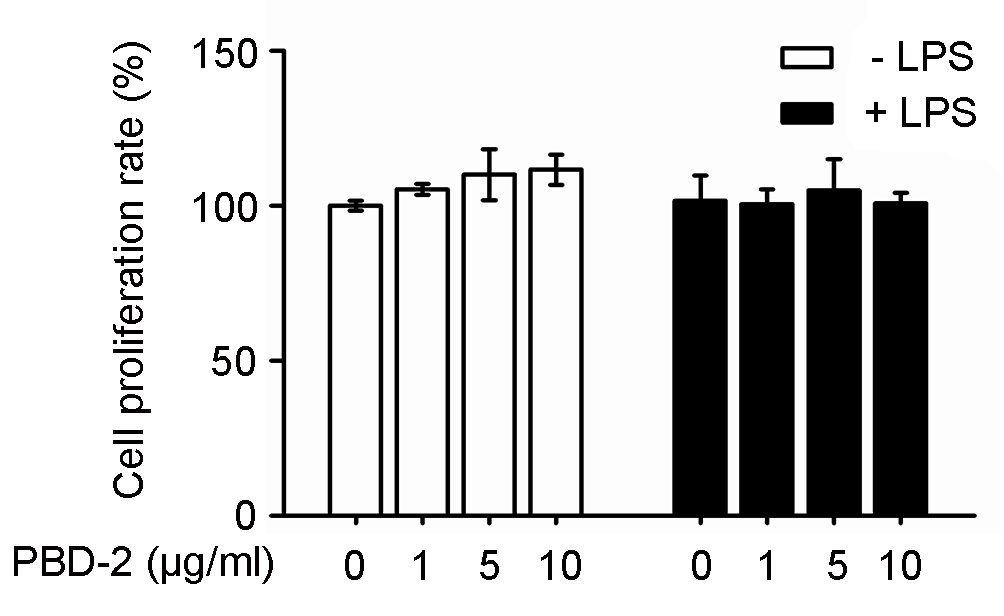

Supplement: Figure S2 — Effect of PBD-2 on the viability of RAW 264.7 cells. Cell proliferation rate was detected by MTT assay. RAW 264.7 cells were incubated with PBD-2 at 1, 5, and 10 μg/ml alone or together with LPS (1 μg/ml). Data are represented as means ± SD (n = 6 each group). [file Image_2.TIF]

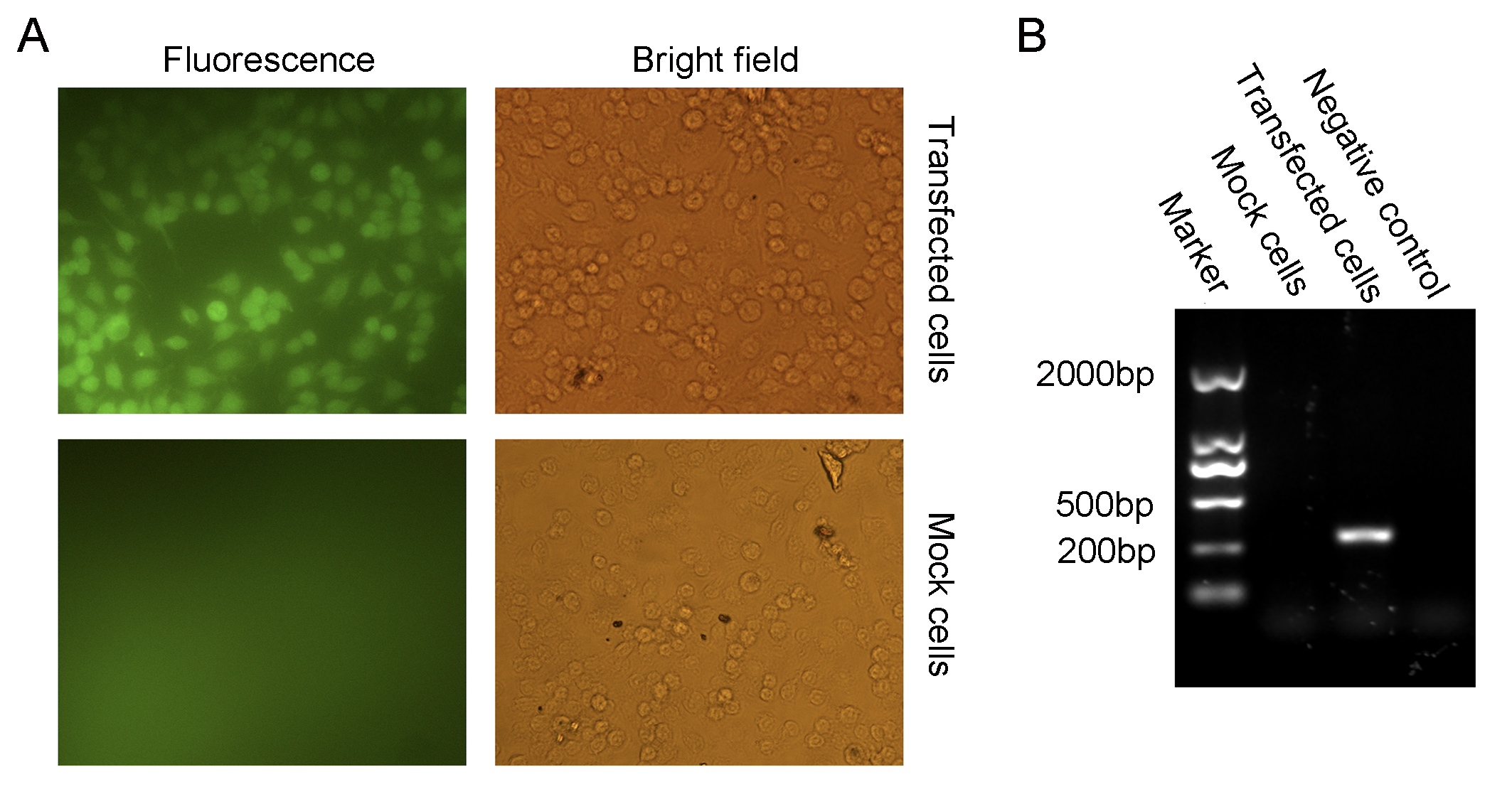

Supplement: Figure S3 — PBD-2 expressing RAW 264.7 cells were confirmed through indirect IFA by using the antiserum of PBD-2 (A) and qRT-PCR by using the PBD-2 detection primers (B). [file Image_3.TIF]

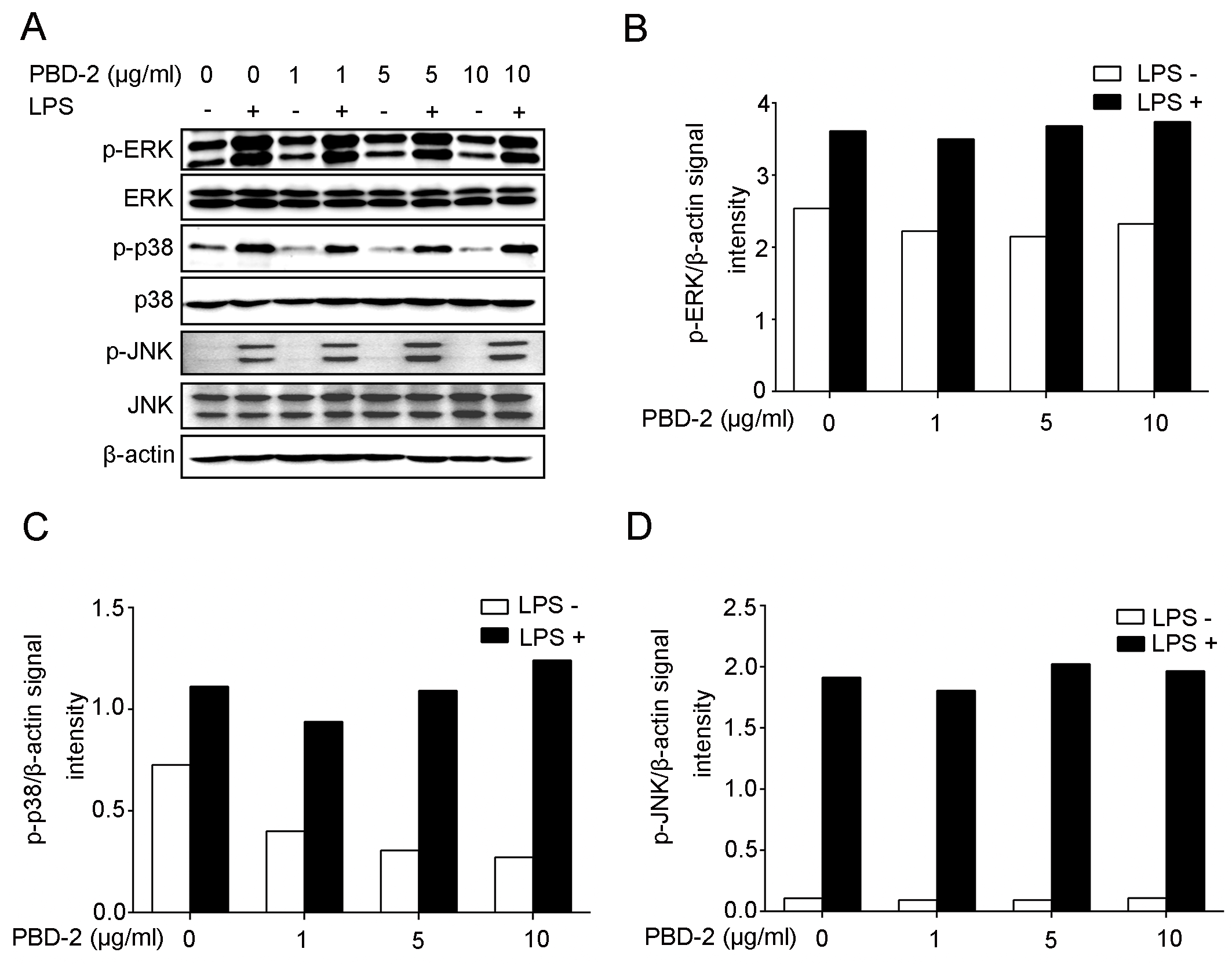

Supplement: Figure S4 — Effects of PBD-2 on ERK, p38, and JNK signaling pathways. Cells were treated with LPS for 30 min with or without various concentrations of PBD-2. The expression levels of ERK, p-ERK, p38, p-p38, JNK, p-JNK, and β-actin were examined by Western blot (A). The signal intensity of each band was quantified (B–D). [file Image_4.TIF]

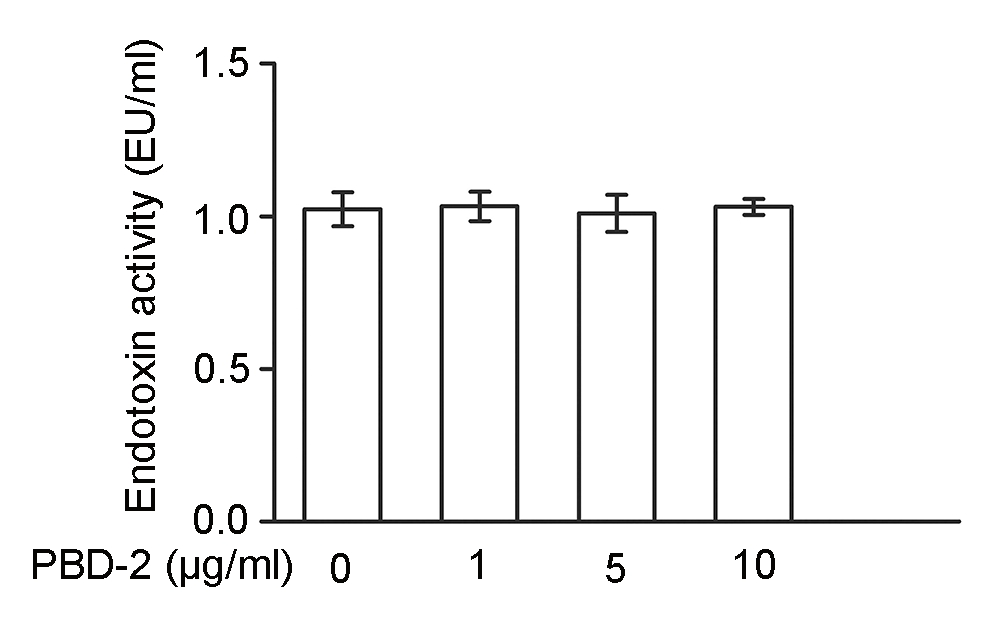

Supplement: Figure S5 — Effect of PBD-2 on the endotoxin activity. Endotoxin activity was detected via a LAL assay. Cells were treated with LPS (1 μg/ml) with or without various concentrations of PBD-2 (1, 5 and 10 μg/ml). Data are represented as means ± SD (n = 6 each group). [file Image_5.TIF]

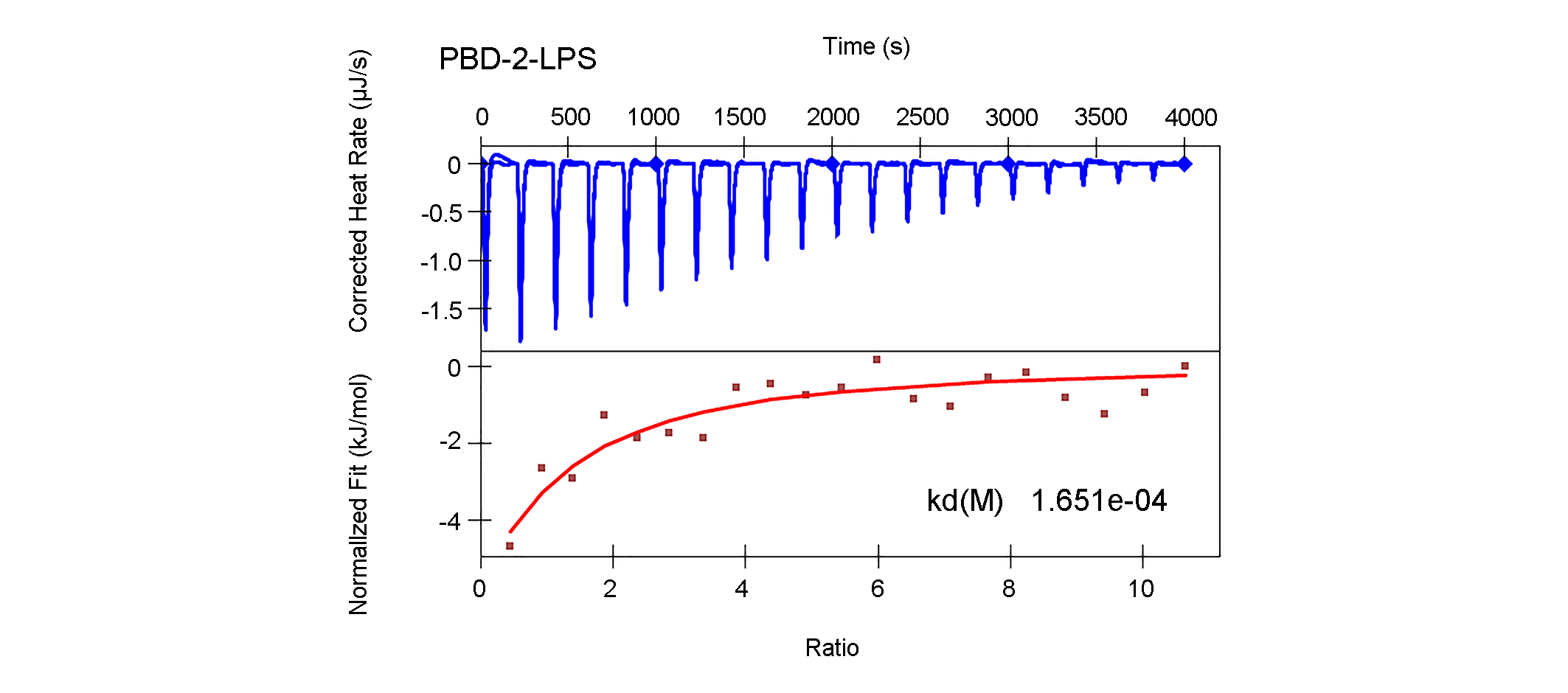

Supplement: Figure S6 — Interaction of PBD-2 with LPS detected by ITC. PBD-2 was placed in a syringe (concentration = 0.75 mM) and LPS was added in the calorimeter cell (concentration = 0.050 mM). PBS was placed in the calorimeter cell as a negative control. [file Image_6.TIF]
