# Supplementary material for: Porcine Beta-Defensin 2 Provides Protection Against Bacterial Infection by a Direct Bactericidal Activity and Alleviates Inflammation via Interference With the TLR4/NF-κB Pathway
Source: Front Immunol. 2019 Jul 18;10:1673. doi: 10.3389/fimmu.2019.01673 (PMC6657668; doi:10.3389/fimmu.2019.01673)
Supplement: Table S1 — Assays of bacterial infection or LPS treatment of mice. [file Table_1.DOCX]

**Table S1**: Assays of bacterial infection or LPS treatment of mice.

| **Aims** | **Groups** | **Inoculation material, dose and route** | **Detection time** |
| --- | --- | --- | --- |
| Fatality rate | TG mice (n = 10) | *S.* typhimurium  (5×10^8^ CFU)  Orally | 15 dpi |
|  | WT mice (n = 10) |  |  |
| Body weight loss | TG mice (n = 7) | *S.* typhimurium (2.5×10^8^ CFU)  Orally | 1, 2, 3, 4 and 5 dpi |
|  | WT mice (n = 7) |  |  |
| Organ weight ratios, bacterial loads in tissues and histopathology analysis of cecum. | TG mice (n = 8) | *S.* typhimurium (2.5×10^8^ CFU)  Orally | 24 hpi |
|  | WT mice (n = 9) |  |  |
| Cytokine detections  (IL-6, TNF-α, IL-1β, IL-12) | WT mice (n = 6) | *S.* typhimurium (2.5×10^8^ CFU)  Orally | 24 hpi |
|  | TG mice (n = 6) |  |  |
|  | WT mice (n = 6) | *S.* typhimurium (2.5×10^8^ CFU)  PBD-2  (5 mg/kg bw)  Orally |  |
|  | WT mice (n = 6) | LPS (16mg/kg bw) Intraperitoneally | 1 hpi |
|  | TG mice (n = 6) |  |  |
|  | WT mice (n = 6) | LPS (16 mg/kg bw)  PBD-2 (0.5 mg/kg bw) Intraperitoneally. |  |

Note: dpi: day(s) post infection; hpi: hour(s) post infection; bw: body weight.

In each experiment, PBS were used as inoculation material on 6 mice as negative control.
