# Supplementary material for: Porcine Beta-Defensin 2 Provides Protection Against Bacterial Infection by a Direct Bactericidal Activity and Alleviates Inflammation via Interference With the TLR4/NF-κB Pathway
Source: Front Immunol. 2019 Jul 18;10:1673. doi: 10.3389/fimmu.2019.01673 (PMC6657668; doi:10.3389/fimmu.2019.01673)
Supplement: Table S2 — Physiological and biochemical indexes of the blood sample from TG and WT mice. [file Table_2.DOCX]

**Table S2:** Physiological and biochemical indexes of the blood sample from TG and WT mice.

| Items | Unit | Concentrations (Mean ± SEM) | |
| --- | --- | --- | --- |
|  |  | TG mice | WT mice |
| ALT | U L^-1^ | 30.00 ± 0.58 | 32.33 ± 2.33 |
| AST | U L^-1^ | 138.77 ± 5.84 | 134.33 ± 11.16 |
| ALT/AST |  | 0.22 ± 0.01 | 0.25 ± 0.03 |
| ALP | U L^-1^ | 135.67 ± 22.85 | 133.20 ± 11.23 |
| GGT | U L^-1^ | 2.10 ± 0.06 | 1.17 ± 0.09 |
| TP | g L^-1^ | 60.77 ± 0.62 | 61.60 ± 0.35 |
| ALB | g L^-1^ | 35.40 ± 0.53 | 35.37 ± 0.81 |
| GLB | g L^-1^ | 25.37 ± 0.09 | 26.23 ± 1.13 |
| A/G |  | 1.40 ± 0.02 | 1.36 ± 0.09 |
| TBIL | μmol L^-1^ | 1.73 ± 0.19 | 1.33 ± 0.59 |
| DBIL | μmol L^-1^ | 0.23 ± 0.09 | 0.37 ± 0.12 |
| Urea | mmol L^-1^ | 12.19 ± 0.48 | 11.58 ± 0.79 |
| Cr | μmol L^-1^ | 33.63 ± 6.05 | 32.67 ± 3.37 |
| U/Cr |  | 0.38 ± 0.06 | 0.37 ± 0.058 |
| Glu | mmol L^-1^ | 9.73 ± 0.79 | 9.23 ± 0.46 |
| K | mmol L^-1^ | 8.70 ± 0.70 | 6.70 ± 0.29 |
| Na | mmol L^-1^ | 158.43 ± 1.15 | 154.97 ± 0.87 |

*ALT* alanine aminotransferase, *AST* aspartate aminotransferase, *GGT* Glutamyl transpeptidase, *TP* treponema pallidum, *ALB* albumin, *GLB* globin, *A/B* albumin/ globin, *TBIL* total bilirubin, *DBIL* direct bilirubin, *UREA* urea, *Cr* creatinine, *U/Cr* urea/ creatinine, *GLU* glucose, *K* kalium, *Na* natrium.
